# Associations of cardiovascular risk factors in Al Ain- United Arab Emirates

**DOI:** 10.1186/1475-2840-8-21

**Published:** 2009-04-16

**Authors:** Latifa M Baynouna, Anthony D Revel, Nico JD Nagelkerke, Tariq M Jaber, Aziza O Omar, Nader M Ahmed, Mohammad K Nazirudeen, Mamdouh F Al Sayed, Fuad A Nour, Sameh Abdouni

**Affiliations:** 1 Al Ain Primary Health Care, Eastern Region Abu Dhabi Emirate, Al-Ain, United Arab Emirates; 2 Department of Community Medicine, Faculty of Medicine and Health Sciences, UAE University, Al-Ain, United Arab Emirates

## Abstract

**Background:**

Over the last 30 years the citizens of the United Arab Emirates have experienced major changes in life-style secondary to increased affluence. Currently, 1 in 5 adults have diabetes mellitus, but the associations (clustering) among risk factors, as well as the relevance of the concept of the metabolic syndrome, in this population is unknown.

**Aim:**

To investigate the prevalence and associations among cardiovascular risk factors in this population, and explore to what extent associations can be explained by the metabolic syndrome according to ATP-III criteria.

**Method:**

A community based survey, of conventional risk factors for cardiovascular disease was conducted among 817 national residents of Al Ain city, UAE. These factors were fasting blood sugar, blood pressure, lipid profile, BMI, waist circumference, smoking, or CHD family history. Odds ratios between risks factors, both unadjusted and adjusted for age and sex as well as adjusted for age, sex, and metabolic syndrome were calculated.

**Results:**

Various risk factors were positively associated in this population; associations that are mostly unexplained by confounding by age and sex. For example, hypertension and diabetes were still strongly related (OR 2.5; 95% CI 1.7–3.7) after adjustment. An increased waist circumference showed similar relationship with hypertension (OR 2.3; 95% CI 1.5–3.5). Diabetes was related to an increased BMI (OR 1.5; 96% CI 1.0–2.3). Smoking was also associated with diabetes (OR 1.9, 95% CI 1.0–3.3).

Further adjustment for metabolic syndrome reduced some associations but several remained.

**Conclusion:**

In this population risk-factors cluster, but associations do not appear to be explained by the presence/absence of the ATP-III metabolic syndrome. Associations provide valuable information in planning interventions for screening and management.

## Introduction

The global burden of disease and mortality due to cardiovascular diseases is huge, and in this respect the United Arab Emirates (UAE) is no exception [1]. Much of this burden is preventable, however, as many of the risk factors associated with these diseases, such as smoking, dyslipidaemia, hypertension etc. have been identified and are amenable to targeted pharmacological and/or behavioural interventions. Associations (i.e. correlations in the presence) and interactions (e.g. the risk associated with obesity may differ between smokers and non-smokers) between these risk factors have been reported in different studies [2-5]. Recognition of such associations and interactions among different risk factors is important in the prevention and management of cardiovascular disease and its complications, as well as in providing a better understanding of the aetiology of these disorders. Any concentration of high CVD risk factors among an identifiable sub-group of the population has obvious implications for prevention strategies.

Certain combinations of risk factors, often called the "metabolic syndrome", have been linked to poorer long-term outcomes. Although, both the existence of this syndrome, its precise definition, and its prognostic relevance have been hotly debated in recent years, it is clear that combinations of risk factors are associated with poorer outcomes [6-8] yet the precise nature of these interactions and associations among risks factors remains moot [9-11].

While many studies have been carried out to establish how to reduce and treat cardiovascular risk factors efficiently, both separately and in combination, [12-15] the majority of these intervention studies were conducted in the Western world, and have an unclear relevance to those parts of the world, with different ethnic and genetic backgrounds and lifestyles, such as the UAE. Lacking regionally equivalent studies the UAE has had to adopt or adapt Western approaches without verification. While several studies on cardiovascular risk factors have been conducted in the UAE, [16,17] none have reported how these risk factors relate and interact.

An important factor that needs to be taken into account in the UAE is ethnicity. The UAE is currently one of the most ethnically diverse countries in the world. Eighty percent of its population consists of non-national (expatriate) migrant workers (Indians, Pakistanis, other Arabs, Europeans,), and even within Nationals (the subjects of our study) there exists a great diversity, as the tribal structure reflects a wide variety of ethnic origins. The importance of ethnicity and the life-style and environment that comes with it is apparent from its association with risk factors such as diabetes. For example, the prevalence of diabetes in Oman was reported to be 16.1% [18] and in Yemen to be between 6.5% and 10.4% [19] respectively. Both of these figures are significantly lower than that found and reported earlier in this study of the same ethnic groups living in a more westernised environment, *viz*. 23.3% [1]. The effect of immigration into a Western lifestyle has been reported in other studies. An example being Yemeni Jews immigrating to Israel who developed high cardiovascular risk profiles [20].

This study is the first community based epidemiological study in the UAE to investigate the major risk factor associations and how cardiovascular risk factors behave in an Arab population with a high cardiovascular risk profile.

## Methods

A cross-sectional community based study was conducted involving 817 UAE citizen subjects.

The methodology of sampling and data collection has been described in detail elsewhere [1]. All continuous risk factor variables were dichotomized to reflect either "normal" or recognized high risk values. Specifically, diabetes was defined as either self-reported diabetes or FBS > = 126 mg/dl, central obesity was defined as waist circumference > 102(m)/88(f) cm, hypertension as BP > = 140/90 or being on antihypertensive medication, obesity as BMI > = 30 kg/m^2^, high TG as > = 150 mg/dl, high LDL > 160 mg/dl and low HDL as < 40 mg/dl.

Odds ratios and their 95% CIs were used to express the strength of associations. In addition to unadjusted odds ratios, odds ratios adjusted for age and sex using the Mantel-Haenszel methodology, and those adjusted for age, sex, and the presence/absence of metabolic syndrome (ATP-III) are presented. The difference between the two adjusted odds ratios supposedly reflects the extent to which the metabolic syndrome "explains" associations among risk factors.

## Results

Table 1 shows associations between key cardiovascular risk factors, using (Mantel-Haenszel) odds ratios (OR), *viz*. BMI, waist circumference, hypertriglycidaemia, low HDL, high LDL, Diabetes, hypertension, smoking, and family history of coronary heart disease in a first degree relative. Associations between risk factors were then adjusted for age and sex to remove the effect of obvious confounders (Table 2). After adjusting for age and sex, hypertension and diabetes remain strongly related with an OR of 2.5 (95% CI: 1.7–3.7; p < 0.00001). Obesity and hypertension showed a similar relationship with an OR of 2.3 (95% CI: 1.5–3.5; p < 0.001) with increased waist circumference. About 50% of the hypertensive patients are obese with the prevalence of hypertension increasing with increasing BMI from 15% in those with BMI less than 25 Kg/m^2 ^to 40% in those with BMI equal or more than 40 Kg/m^2^.

**Table 1:** The raw associations of the different risk factors studied

Diabetes	4.1							
	2.8–5.8							
						
Central Obesity	2.2	1.3						
	1.5–3.2	0.9–1.8						
					
Obesity	2	1.1	15.9					
	1.4–2.8	0.8–1.6	10.5–23.8					
				
Smoking	1	1.3	0.8	0.8				
	0.5–1.7	0.8–2.1	0.5–1.2	0.5–1.3				
			
High TG*	1.7	1.6	1.4	1.1	1.2			
	1.2–2.4	1.2–2.3	1.0–1.9	0.8–1.5	0.8–2.0			
		
High LDL	1	0.6	0.9	1.2	1	1		
	0.6–1.5	0.4–1.0	0.6–1.4	0.9–1.8	0.5–1.7	0.7–1.4		
	
Low HDL	1.6	1.5	0.9	1.1	2.6	1.6	1.2	
	1.1–2.2	1.1–2.1	0.7–1.3	0.8–1.1	1.6–4.2	1.2–2.2	0.9–1.7	
Family history of CHD	0.8	0.7	1.3	1.8	2.4	1	1.4	1.2
	0.5–1.4	0.4–1.2	0.8–2.0	1.1–2.7	1.4–4.3	0.6–1.6	0.8–2.3	0.8–1.8

	Hypertension	Diabetes	Central Obesity	Obesity	Smoking	High TG	High LDL	Low HDL

**Table 2:** Odds ratios of the associations among different cardiovascular risk factors adjusted for age and sex, using Mantel-Haenszel's method

Diabetes	2.5							
	1.7–3.7							
						
Central Obesity	2.3	1.2						
	1.5–3.5	0.8–1.7						
					
Obesity	2.5	1.5	15.2					
	1.6–3.6	1.0–2.3	9.7–23.8					
				
Smoking	1.5	1.8	1.2	1.1				
	0.8–3.0	1.0–3.3	0.7–2.1	0.7–1.9				
			
High TG	1.6	1.5	1.5	1.1	1.2			
	1.1–2.3	1.1–2.2	1.0–2.1	0.8–1.5	0.7–1.9			
		
High LDL	0.8	0.5	1	1.4	0.9	1		
	0.5–1.3	0.3–0.8	0.6–1.5	0.9–2.0	0.5–1.6	0.7–1.5		
	
Low HDL	1.6	1.4	1.1	1.3	1.8	1.5	1.1	
	1.1–2.4	1.0–2.0	0.8–1.5	0.9–1.8	1.1–3.1	1.1–2.0	0.8–1.6	
Family history of CHD	0.9	0.9	1.2	1.6	3	1	1.5	1.3
	0.5–1.7	0.5–1.6	0.8–1.9	1.0–2.5	1.5–6.0	0.7–1.6	0.9–2.5	0.8–2.0

	Hypertension	Diabetes	Central Obesity	Obesity	Smoking	High TG	High LDL	Low HDL

**Figure 1:**
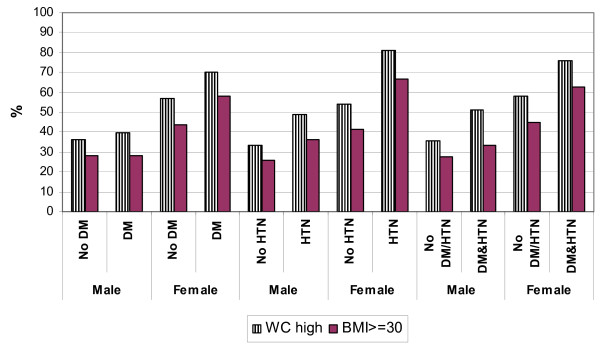
**Waist circumference and BMI related to diabetes, hypertension and those with both diabetes and hypertension**.

Similarly, an increasing BMI was associated with diabetes with the OR for the association between diabetes and obesity (BMI > = 30) being 1.5 (95% CI : 1.0–2.3), but the association with diabetes was somewhat weaker and not statistically significant for increased waist circumference (as opposed to BMI), with an OR of 1.2 (95% CI : 0.8–1.7). Figure 1 shows the relationship between an increasing BMI and waist circumference on one hand, with diabetes and hypertension on the other.

Dyslipidaemia was differently related to hypertension and diabetes. A significant association was found between hypertension and increased TG and decreased HDL, with ORs of 1.6 (95% CI: 1.1–2.3; p = 0.02) and 1.6 (95% CI: 1.1–2.4, p = 0.01) respectively.

Associations for diabetes demonstrated a weak, but statistically significant, association with increasing TG [OR of 1.5 (95% CI: 1.1–2.2)], an interesting, inverse relationship, was seen with LDL with an OR of 0.5(95% CI: 0.3–0.8; p = 0.001) i.e. diabetes was found to be associated with low LDL.

In the case of dyslipidaemia and measures of obesity the only association was between increased waist circumference and high TG, with an OR of 1.5 (95% CI: 1.0–2.1). There was no association between waist circumference and high LDL or low HDL, nor did dyslipidaemia parameters show any significant associations with an increased BMI.

Smoking showed an interesting strong relationship with a history of premature IHD in first degree relative OR 3 (95% CI: 1.5–6; p = 0.001). A weak association was noticed as well between high BMI and a history of premature IHD in a first degree relative OR 1.6 (1–2.5) but it did not reach statistical significance (p = 0.4).

Another interesting finding was that diabetes and smoking are significantly related, with an OR of 1.8 (95% CI: 1.0–3.3).

Table 3 represents associations after adjusting for the metabolic syndrome. Although many associations were reduced there remained a significant association between obesity and high LDL with an OR of 1.7 (95% CI: 1.1–2.5).

**Table 3:** Odds ratios of the associations among different cardiovascular risk factors adjusted for Age, sex and ATP defined metabolic syndrome

Diabetes	1.5							
	0.9–2.2							
						
Central Obesity	1.2	0.4						
	0.7–2.0	0.2–0.7						
					
Obesity	1.9	0.9	16.1					
	1.2–3.0	0.6–1.4	9.5–27.3					
				
Smoking	1.4	1.5	0.9	0.8				
	0.7–2.9	0.8–2.8	0.5–1.7	0.5–1.5				
			
High TG	0.7	0.6	0.4	0.6	10.9			
	0.5–1.1	0.4–0.9	0.2–0.7	0.4–0.8	0.5–1.7			
		
High LDL	1	0.5	1.3	1.7	0.9	1.4		
	0.6–1.6	0.3–0.9	0.8–2.0	1.1–2.5	0.5–1.6	0.9–2.2		
	
Low HDL	1.1	0.7	0.4	1	1.6	0.7	1.3	
	0.7–1.6	0.4–1.0	0.3–0.7	0.7–1.3	0.9–2.8	0.5–1.1	0.9–2.0	
Family history of CHD	0.9	0.8	0.9	1.5	2.7	0.9	1.6	1.1
	0.5–1.6	0.4–1.5	0.5–1.5	0.9–2.3	1.3–5.5	0.5–1.6	0.9–2.7	0.7–1.9

	Hypertension	Diabetes	Central Obesity	Obesity	Smoking	High TG	High LDL	Low HDL

**Figure 2:**
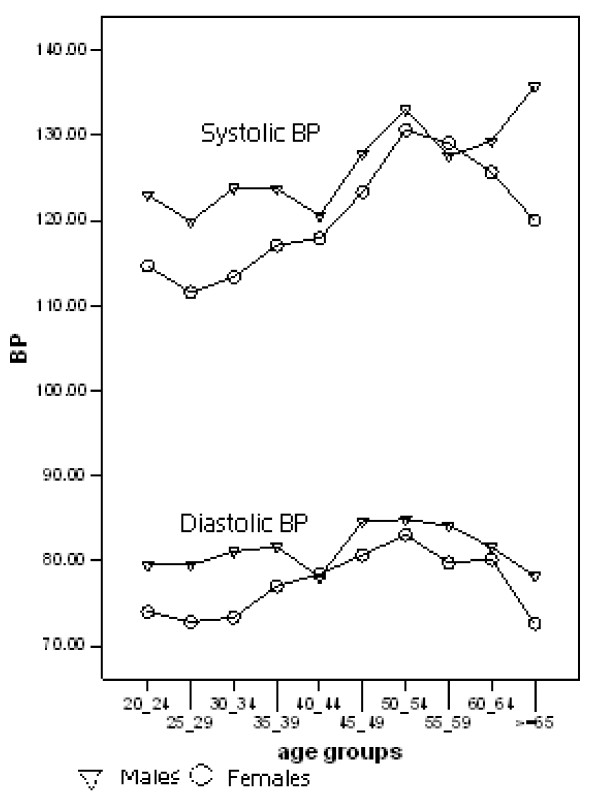
**The Gender influence on blood pressure and the change of blood pressure with age**.

Blood pressure was related to gender (Figure 2), with females having lower systolic and diastolic blood pressure than males, the difference decreasing with age.

**Figure 3:**
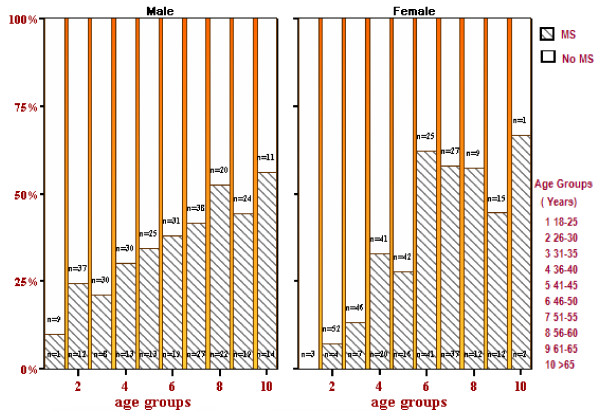
**The prevalence of Metabolic Syndrome**.

Figure 3 shows the change in prevalence with age and sex of the metabolic syndrome (using ATP III criteria [21]). It reaches a prevalence of 37% in males around 50 years old and exceeds 50% in females between 45 and 50 years. Overall prevalence was 37.6% more than that reported in our previous report (22.7%) [1] since in the former percentage diabetics and self-reported (treated) hypertensives were included as having IGT and high blood pressure respectively.

Ethnicity and its relationship to risk factors was studied by inquiring about subjects' tribe name, place of birth and their self-identified origins. Analysis by tribes was complicated by the large number of tribes giving rise to small sample sizes per group. Subjects were therefore grouped to (a) originally from UAE and born in the UAE, (b) originally from Yemen, (c) originally from Oman, and (d) other origins. There appeared to be no striking associations between ethnicity and prevalence of risk factors, with the exception that subjects originally from Yemen tended to have a somewhat higher BMI, with an OR for obesity of 0.6 (95% CI 0.4–0.9).

## Discussion

The study revealed some important associations among CVD risk factors, which persisted after correcting for the confounders of age and gender. Adjusting for the metabolic would be expected to nullify most of these associations as the syndrome is defined in terms of the same risk factors. Despite this several associations persisted after that adjustment such as those between hypertension and obesity and between high LDL and obesity. The implications of these findings is that the premise of a simple uni-dimensional (metabolic) syndrome, at least not as defined by the ATP III criteria, cannot be applied to the population studied.

The population of Al Ain, as in many parts of the world and like other Arabian Gulf states, has experienced a major shift in lifestyle that has been accompanied by a proliferation of cardiovascular risk factors [1]. In this Arab population the prevalence and association among different risk factor interactions is worth reporting as it provides information for patients and health care policy makers to prevent and manage the problem with targeted interventions.

A recent meta-analysis to determine the best index of obesity examined the association between diabetes and the three indices of body mass index, waist circumference, and waist/hip ratio. The findings were inconclusive to determine the usefulness of waist circumference or waist/hip ratio over body mass index [22]. In this study hypertension was correlated with both increasing BMI and increasing waist circumference while diabetes correlated predominantly with increased BMI. In a study done on Asians, Waist-to-Stature Ratio was a stronger correlate than BMI of diabetes, but these two indicators of obesity were equally strongly associated with hypertension [23]. A meta-analysis has supported this finding in the case of hypertension [24].

Our finding of dyslipidaemia (increased triglycerides and low HDL) being associated with diabetes has been shown in other studies [25] but in this study diabetes was paradoxically associated with low LDL. This could be due to lipid lowering medication or the effect of lifestyle changes. This paradoxical association was not true for hypertension nor for diabetes and hypertension combined in the same individual.

There is a strong association between smoking and family history of premature CHD and between increased BMI and family history of premature CHD. This could be explained by a genetic predisposition to develop risk factors for cardiovascular diseases but may equally be due to clustering of adverse lifestyle choices within families. This needs to be explored in greater detail. Interestingly, our study corroborates the association between diabetes and smoking that was reported in a number of studies, indicating that cigarette smoking is an independent and -of course- modifiable determinant of type 2 diabetes mellitus. Regrettably, we did not measure passive smoking, so we could not confirm the association of DM with passive exposure to cigarette smoke that has also been reported [26-28].

Blood pressure showed about 10 mmHg differences between males and females in the younger age group, being higher in males, but this difference diminished with age. In the age group above 60 years it diverts again but no firm conclusions can be reached in view of the small sample sizes in the older age groups. Our findings largely coincide with a study conducted in Japan [29].

Many studies have identified the association of diabetes and hypertension, including one conducted in nearby Saudi Arabia [30]. As both are influenced by obesity our findings of a similar association is to be expected. The precise causal pathway among these variables cannot be established in our cross-sectional study.

Metabolic syndrome allegedly describes a cluster of cardiovascular risk factors. This study failed to find a clear association between the different metabolic syndrome criteria in the individuals diagnosed with the syndrome. This corresponds with other recent reports that have used factor analysis to investigate these associations [28-30]. This would suggest that the causal link between these diagnostic components may not be sufficiently strong to justify the concept of a "syndrome". However, statistical criteria should not have the last word and it may be necessary to study other pathological changes reported in relation to the metabolic syndrome to investigate if a unique syndrome exists. In addition, the suggested prognostic value of this syndrome was found to be variable depending on the specific definition used [31]. This clearly indicates that it needs to be further explored in order to determine whether its prognostic value exceeds that of the (multiplicative) Framingham risk score which simply treats CVD risk empirically without any reference to nosological concepts or hypotheses.

## Competing interests

The authors declare that they have no competing interests.

## Authors' contributions

LMB, ADR, TMJ, NMA, MKN, FAN, MFA participated in the study design. NMA, MKN, FAN, MFA, SA carried out the study in the centres. LMB and AOO coordinated the study. NJDN performed the statistical analysis. LMB, ADR, NJDN drafted the manuscript. All authors read and approved the final manuscript.
